# Comparative immunological roles of TEP1 in *Anopheles gambiae* and *Biomphalaria glabrata*: implications for malaria and schistosomiasis control

**DOI:** 10.3389/fimmu.2025.1629262

**Published:** 2025-10-15

**Authors:** Hongyu Li, Yilu Feng, Yuncheng Qian, Wenjie Jiang, Yunhuan Zhu, Jialu Xu, Xianwei Li, Xinyi Fei, Ruke Wang, Yuqing Shao, Lailing Du, Xiaofen Zhang, Keda Chen

**Affiliations:** Key Laboratory of Artificial Organs and Computational Medicine in Zhejiang Province, Shulan International Medical College, Zhejiang Shuren University, Hangzhou, China

**Keywords:** *Anopheles gambiae*, immune, plasmodium, schistosomamansoni, thioester-containing protein

## Abstract

Malaria and schistosomiasis represent two of the most significant global parasitic diseases in terms of public health burden. These diseases are transmitted through *Anopheles* mosquitoes and freshwater snails, respectively. Although their transmission mechanisms differ, both pathogens critically interact with thioester-containing proteins (TEPs) during immune evasion and clearance within their invertebrate hosts. This review compares the activation mechanisms and functional divergences of TEPs in *Anopheles gambiae* and *Biomphalaria glabrata* in the context of host anti-infective immunity. We focus on the roles of *Ag*TEP1 and *Bg*TEP1 in pathogen opsonization and elimination, discussing their interaction networks with co-factors such as LRIM1/APL1C, *Bg*FREPs and Biomphalysin. Furthermore, we analyze differences in immune pathways mediated by TEPs, including reactive oxygen species (ROS) generation, phagocytic elimination, and melanization responses, as well as their regulatory mechanisms governed by host genetic backgrounds and environmental factors. The review also evaluates the evolutionary roles of TEPs in host-parasite coevolution and highlights their potential application in vector intervention and disease prevention strategies. By elucidating both conserved and species-specific characteristics of the TEP system in these evolutionarily distant invertebrates, this work provides critical insights into the evolutionary trajectories of invertebrate innate immunity and advances theoretical frameworks for novel vector control approaches.

## Introduction

Parasitic diseases remain one of the most critical public health challenges in developing countries, with malaria and schistosomiasis ranking among the top in terms of incidence rates and mortality, posing severe threats to human health and economic development (1–4).

*Anopheles* mosquitoes, particularly the *An. gambiae* complex, serve as primary malaria vectors in Africa due to their marked anthropophily, high transmission efficiency, and growing insecticide resistance (5, 6). Globally, malaria caused ~247 million cases and 619,000 deaths in 2021, with >95% occurring in Africa (7). While artemisinin-based combination therapies (ACTs) remain first-line treatment, emerging evidence of parasite resistance underscores the urgent need for novel interventions (8).

Schistosomiasis is another global parasitic disease caused by trematode worms of the genus *Schistosoma*, affecting over 250 million people and predominantly endemic in tropical and subtropical regions (4, 9). Among these, *Schistosoma mansoni* stands as one of the primary etiological agents, completing its life cycle through freshwater snails (e.g., *Biomphalaria* spp.) as intermediate hosts (7, 10). Despite praziquantel (PZQ) being the drug of choice, its inability to prevent reinfection and reports of reduced efficacy highlight critical limitations for its use (11, 12).Consequently, targeting and reducing the infection rates of intermediate host snails has become a critical strategy for interrupting schistosomiasis transmission.

Despite the disparities in parasite taxonomy and transmission mechanisms between these two diseases, their life cycles fundamentally depend on specific invertebrate hosts. These hosts not only provide essential developmental niches for the parasites but also influence their survival and transmissibility through sophisticated immune mechanisms.

Thioester-containing proteins (TEPs) – immune effectors homologous to and structurally similar to vertebrate complement components – have emerged as a research focus in invertebrate immunology due to their central roles in pathogen recognition, opsonization, and clearance (13, 14).In *An. gambiae*, *Ag*TEP1 represents the most extensively studied TEP member. Characterized by a conserved thioester motif (GCGEQ), it covalently binds pathogen surfaces and synergizes with LRIM1 and APL1C to stabilize its conformation, enabling specific recognition of *Plasmodium* ookinetes. This molecular complex orchestrates phagocytic elimination or melanization responses against invading parasites (15, 16). Similarly, in *B. glabrata*, the TEP ortholog *Bg*TEP1 demonstrates analogous functions during *S. mansoni* infections. Beyond pathogen surface binding, it cooperates with fibrinogen-related proteins (FREPs) and Biomphalysin to induce oxidative stress responses and mediate sporocyst damage in *S. mansoni* (10).

Although these two systems demonstrate functional convergence, they exhibit significant divergence in activation patterns, cofactor requirements, cellular origins, and regulatory mechanisms. For instance, *Ag*TEP1 is primarily activated in the hemolymph and stabilized through interactions with specific protein complexes, whereas *Bg*TEP1 is predominantly synthesized in haemocytes, with its expression levels showing marked dependence on genotypic variations and environmental factors (10, 17).

However, a comparative review of TEP-mediated immune mechanisms in these two critical vector species is lacking. Such a comparison would not only advance our understanding of the evolutionary diversity and functional convergence of TEPs but also provide theoretical foundations for developing novel antiparasitic intervention strategies. This review aims to provide a focused and comparative analysis of the immune effector proteins *Ag*TEP1 and *Bg*TEP1 in *An. gambiae* and *B. glabrata*, respectively. By dissecting their activation mechanisms and co-factor interactions, we illustrate how these molecules orchestrate species-specific responses to parasitic infections. Through cross-species comparison, we reveal both conserved and divergent strategies employed by these vectors in recognizing and eliminating parasites, emphasizing the evolutionary and ecological implications of TEP-mediated immunity. Ultimately, this work aims to advance our understanding of invertebrate immune evolution and inform future research into immune modulation strategies for parasite control. Through cross-species comparative studies of TEP systems, we seek to deepen insights into the evolutionary mechanisms of invertebrate innate immunity while identifying novel targets for vector control and parasitic disease management. These efforts hold heightened significance given the escalating challenges of drug resistance and the current limitations in vaccine accessibility (11).

## Malaria and its *Anopheles* mosquito vector

Malaria is primarily caused by five *Plasmodium* species infecting humans: *P. falciparum*, *P. vivax*, *P. malariae*, *P. ovale*, and *P. knowlesi* (18, 19). These protozoan parasites, classified under the phylum *Apicomplexa*, exhibit complex life cycles involving two distinct host types: an invertebrate definitive host (where sexual reproduction occurs) and a vertebrate intermediate host. Among them, *P. falciparum* and *P. vivax* are the most prevalent and lethal in humans (20, 21). Notably, *P. falciparum* is responsible for the majority of global malaria related fatalities, with infections characterized by high fever, chills, headache, anemia, hepatosplenomegaly, and severe complications such as renal failure, cerebral malaria, and death.

**Figure 1:**
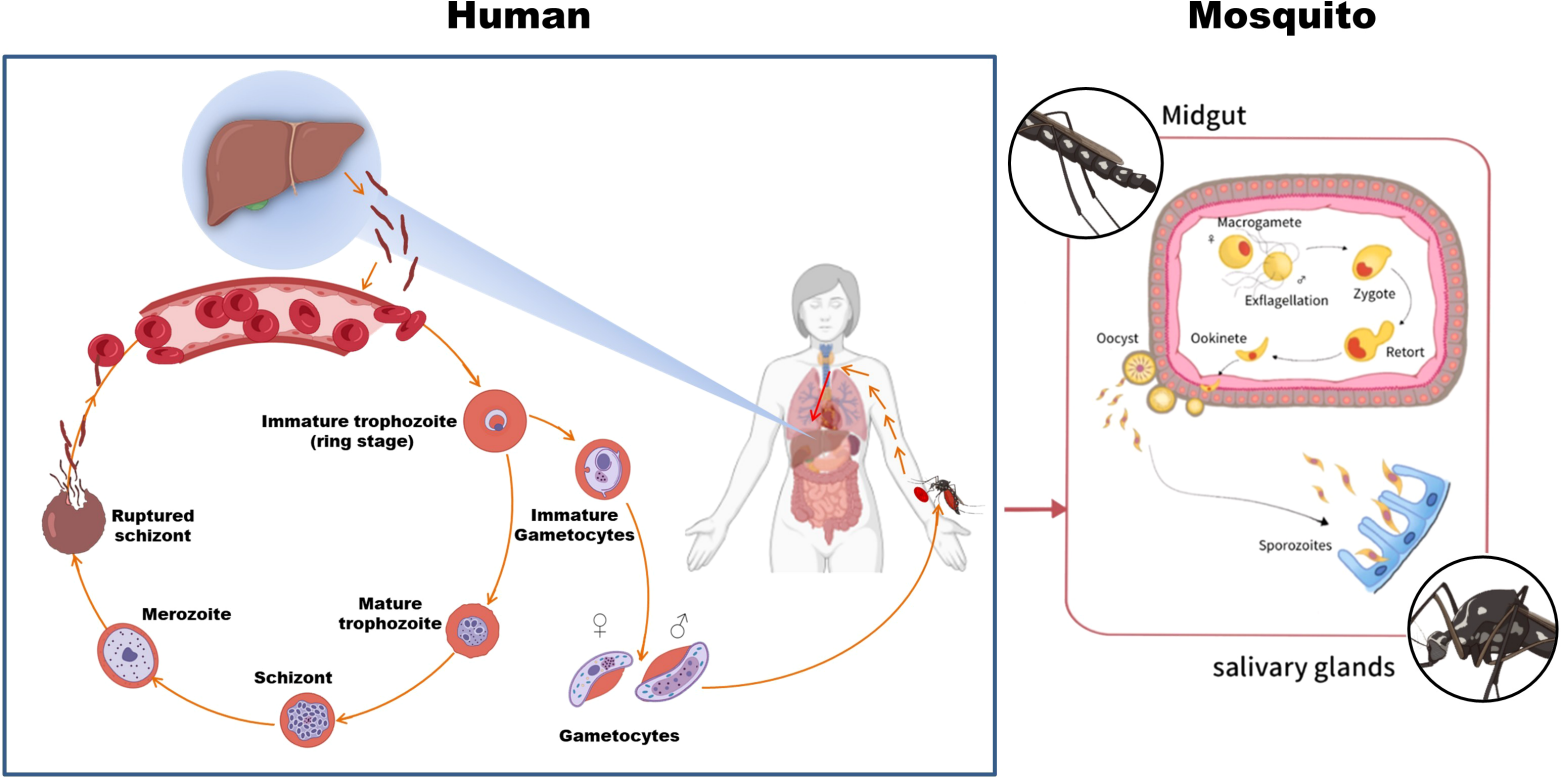
The life cycle of *P. falciparum*. The parasite alternates between human and *Anopheles* mosquito hosts. Sporozoites are transmitted during a mosquito bite, develop in the liver, and invade red blood cells; gametocytes taken up by mosquitoes complete the cycle. Parts of the materials used in this figure originate from BioRender and have been further modified and enhanced.

As illustrated in **Figure 1**, the *P. falciparum* life cycle involves two hosts: humans as intermediate hosts and female *Anopheles* mosquitoes as definitive hosts. During a mosquito bite, sporozoites are injected into the human bloodstream, subsequently migrating to hepatocytes where they undergo hepatic schizogony, producing numerous merozoites. Upon release into the bloodstream, these merozoites invade erythrocytes, initiating the intraerythrocytic cycle marked by sequential developmental stages—ring stages, trophozoites, and schizonts—during which hemoglobin metabolism and asexual replication occur (22, 23). A subset of merozoites differentiates into gametocytes, which, upon ingestion by mosquitoes, undergo sexual reproduction in the mosquito midgut to form zygotes, motile ookinetes, and oocysts. Sporozoites released from mature oocysts migrate to the salivary glands, completing the transmission cycle (24).

*An. gambiae* has emerged as a pivotal model organism for investigating *Plasmodium*-mosquito interaction mechanisms. Its genome has been fully sequenced and annotated, providing a robust foundation for elucidating the interplay between mosquito immune systems and *Plasmodium* parasites (25, 26). Notably, *An. gambiae* s.l. (sensu lato) comprises a complex of morphologically indistinguishable yet genetically and ecologically divergent sibling species (27). *Ag*TEP1 is ubiquitously distributed across this species complex, though its expression levels and spatial-temporal distribution vary significantly among constituent species and geographical populations. Current research on *Ag*TEP1 predominantly focuses on the nominal species *An. gambiae* s.s. (sensu stricto), and the *Ag*TEP1 discussed in this review is derived exclusively from studies on this model taxon, without interspecific distinctions. **Table 1** summarizes the geographical distribution and ecological roles of *An. gambiae* s.l. subspecies in malaria parasite transmission (33).

**Table 1:** The role and geographic distributioN OF MAJOR TAXONOMIC SPECIES Within the *An*.

	*Anopheles* sp.	Impact on malaria transmission and distribution	*Plasmodium* sp. transmitted	Reference
*Anopheles gambiae* s.l.	*Anopheles gambiae* s.s.	the primary vector for malaria in sub-Saharan Africa.	Primarily transmits *P. falciparum*, with potential to spread other malaria parasites.	(28)
	*Anopheles coluzzii*	a significant vector for malaria transmission in the West African region.	Mainly transmits *P. falciparum*.	(29)
	*Anopheles melas*	a key vector for malaria transmission in the Guinea Gulf region.	Mainly transmits *P. falciparum*.	(30)
	*Anopheles quadriannulatus*	a malaria vector with limited impact in certain regions of Africa.	Serves as a vector for various malaria parasites, including *P. falciparum*, *P. vivax*, and *P. ovale*.	(31)
	*Anopheles* arabiensis	a limited role in malaria transmission, but it is capable of contributing to transmission in certain areas.	Mainly transmits *P. falciparum*	(32)

*Gambiae* complex in malaria transmission.

The developmental stages of *Plasmodium* within the mosquito vector are critical to its life cycle, making this phase a prime target for strategies aimed at interrupting malaria transmission. To achieve this goal, a comprehensive understanding of the *Anopheles* innate immune system is paramount. When a mosquito ingests blood containing gametocytes, the parasites must traverse the midgut epithelium, enter the hemocoel, and complete gamete fusion and oocyst formation. Throughout this process, the mosquito orchestrates multifaceted immune responses, including phagocytosis, melanization cascades, and the expression of antimicrobial peptides (AMPs) (10, 34, 35).

Among these immune responses, *Ag*TEP1 functions as a pivotal immune effector by recognizing and binding to *Plasmodium* surfaces via its conserved thioester bond, thereby triggering immune clearance. Functionally resembling vertebrate complement proteins, *Ag*TEP1 mediates pathogen lysis or melanization encapsulation and represents the most extensively characterized TEP to date (18, 20, 21, 36).

## TEP proteins in *An. gambiae*

The TEP family comprises evolutionarily conserved immune molecules widely distributed across invertebrates and vertebrates, including mammals (37). Most TEPs harbor a canonical thioester bond, though this structural motif is absent in certain homologs, such as complement component C5 or specific insect TEPs (13). In vertebrates, TEPs predominantly manifest as components of the complement system (e.g., C3, C4) and serine protease inhibitors like α2-macroglobulin, with their primary function centered on pathogen recognition and elimination (38, 39). In contrast, insect TEPs (often termed iTEPs) have evolved structural and functional diversity through long-term evolutionary processes, exhibiting functional roles analogous to vertebrate α2-macroglobulin (15).

**Figure 2:**
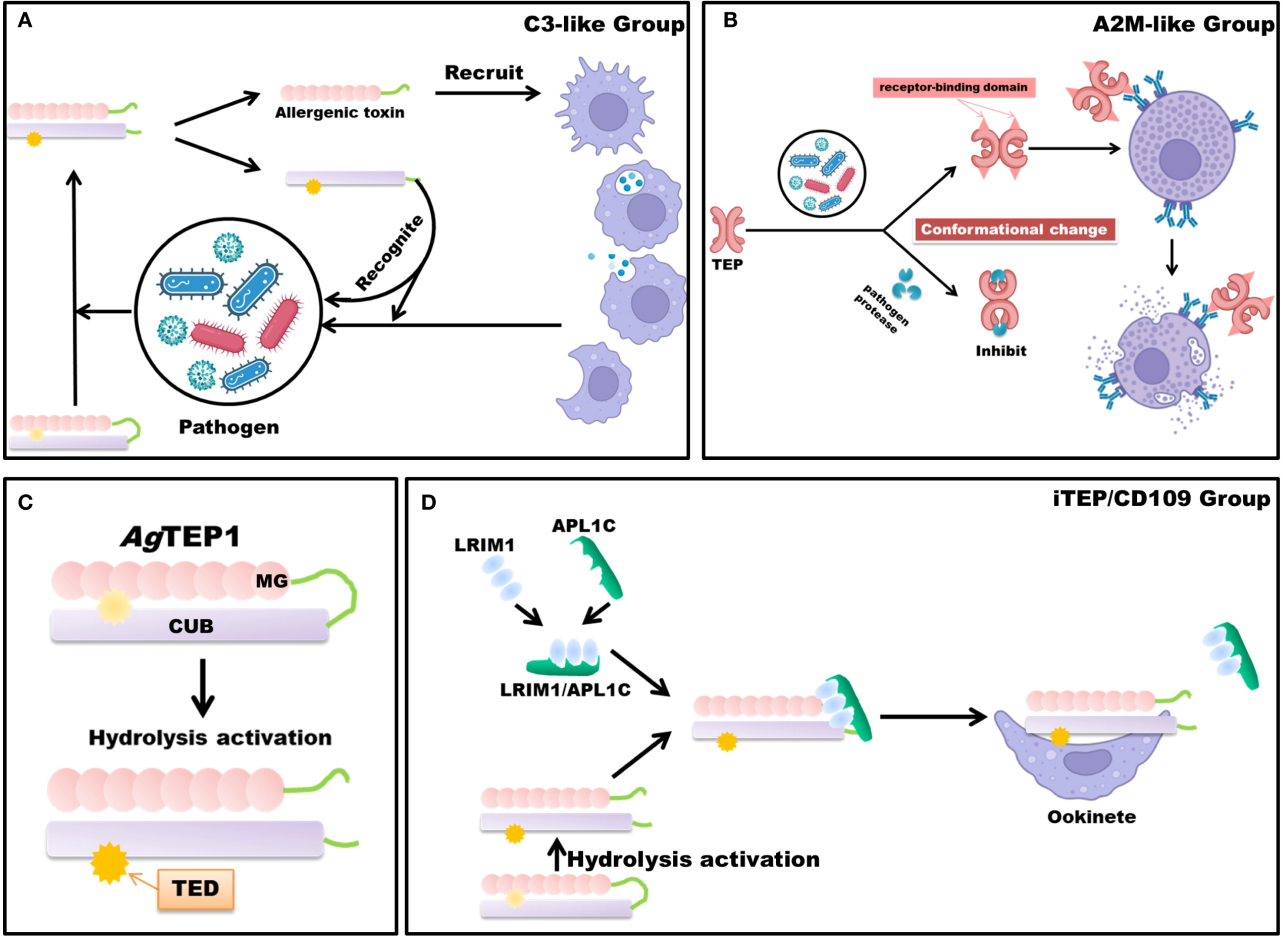
Schematic Diagram of TEP Mechanisms. TEPs are broadly categorized into three principal classes: the iTEP/CD109 group, the complement component C3-like group, and the A2M-like group. **(A)** The mechanism of the complement component C3-like group occurs following immunological stimulation. Upon activation, TEPs undergo proteolytic cleavage, releasing a small allergenic toxin fragment. This fragment acts as an immunostimulant or chemotactic agent, recruiting macrophages to the site of infection. Concurrently, the larger fragment, through covalent bonding via the thioester bond, targets and marks the pathogen, thereby facilitating its degradation or phagocytosis. **(B)** Protease inhibition and immune activation mechanisms of A2M-like TEP. Following engagement with pathogens, A2M-like proteins eschew proteolytic cleavage, undergoing instead a conformational transformation that effectively attenuates the proteolytic activity of the pathogen. In tandem, this conformational rearrangement reveals the receptor-binding domain of A2M, which enhances its interaction with phagocyte surface receptors, thus augmenting endocytosis and expediting pathogen clearance. **(C)** Simplified structure and hydrolytic activation of *Ag*TEP1. **(D)** Members of the iTEP/CD109 group, such as *Ag*TEP1, rely on stabilization mechanisms to prevent premature inactivation after hydrolysis. The LRIM1/APL1C heterodimer binds to hydrolyzed *Ag*TEP1, forming a stable complex that prevents aggregation and ensures functional integrity. This stabilized complex facilitates subsequent immune recognition and binding to invading parasites such as *Plasmodium* ookinetes. Parts of the materials used in the images within this article originate from BioRender, which we have further modified and enhanced.

TEPs are recognized as critical members of the pattern recognition receptor (PRR) family and constitute essential components of the invertebrate innate immune system (40). Cross-species analyses classify TEPs into three major categories: iTEP/CD109-like, C3-like, and A2M-like. In insects, iTEP/CD109-like proteins are typically secreted opsonins and immune modulators that bind pathogen surfaces to promote phagocytosis or melanization; in vertebrates, the homologous CD109 is predominantly a GPI-anchored cell-surface glycoprotein involved in modulation of cell signaling (e.g., TGF-β). This reflects domain-level conservation but functional diversification — with noted exceptions (some invertebrate CD109-like proteins are membrane-associated or processed into soluble forms) (17, 41). C3-like TEPs undergo proteolytic activation into two fragments upon stimulation: the smaller fragment mediates chemotactic and inflammatory signaling, while the larger fragment retains the thioester motif, enabling covalent binding to pathogen surfaces to facilitate clearance (42–44) (**Figure 2A**). A2M and its homologs typically undergo a conformational change (mediated through a bait-trap mechanism) following proteolytic cleavage within their bait regions, thereby entrapping and inhibiting protease activity derived from pathogens or host sources; subsequently, these complexes are cleared via receptor-mediated endocytosis involving low-density lipoprotein receptor-related protein 1 and related receptors (45) (**Figure 2B**).

In *An. gambiae*, the genome encodes at least nineteen *Ag*TEPs (*Ag*TEP1 – 19) that form three broad clades of complement-like factors, with *Ag*TEP1 standing out as the best-studied member to date (45, 46). *Ag*TEP1 is translated as a 165 kDa, N-glycosylated precursor whose architecture follows the canonical C3-like scaffold: eight macroglobulin (MG) domains (MG1-MG8) followed by a CUB domain and a thioester domain (TED) carrying the reactive *GCGEQ* motif. Crystallographic and cryo-EM comparisons reveal a root-mean-square deviation of ≈3 Å between the *Ag*TEP1 core and mammalian C3, confirming close tertiary structural homology (42, 47).

After secretion into the hemolymph, an as-yet-unidentified CLIP-family serine protease cleaves *Ag*TEP1 within the flexible MG6-LNK hinge, generating the disulfide-linked α- chain (75 kDa) and β-chain (85 kDa) that constitute the mature, reactive form *Ag*TEP1-cut. Proteolytic activation unlocks the thioester, allowing covalent attachment to primary hydroxyl or amino groups on microbial surfaces and thereby labelling invaders for downstream immune attack (48, 49) (**Figure 2C**).

Free *Ag*TEP1-cut is intrinsically unstable and tends to precipitate. Two secreted leucine-rich repeat proteins, LRIM1 and APL1C, assemble via a C-terminal coiled-coil into a disulfide-bonded heterodimer that docks one molecule of *Ag*TEP1-cut to form a stable ternary complex. This interaction preserves thioester reactivity, channels *Ag*TEP1 to *Plasmodium* ookinetes or bacteria, and prevents wasteful self-attack. Loss-of-function or RNAi of either LRIM1 or APL1C abolishes TEP1 loading on pathogens and converts refractory mosquitoes into susceptible ones, underscoring the complex as the core of the mosquito complement-like pathway (14, 22–24) (**Figure 2D**).

Recent work further shows that the LRIM1/APL1C carrier can also load other *Ag*TEPs (e.g., *Ag*TEP3) and that non-catalytic cofactors such as SPCLIP1 (a catalytically inactive serine protease-like protein) orchestrate the localized accumulation of *Ag*TEP1 on microbial targets, highlighting a vertebrate-style convertase cascade now being unravelled in insects (44).

Additionally, *Ag*TEP1 exhibits broad-spectrum immune activity by recognizing bacterial pathogens, underscoring its versatility in pathogen surveillance (50).

The immunological efficacy of *Ag*TEP1 displays population specificity, with binding capacity modulated by both host and parasite polymorphisms—a hallmark of host-parasite coevolution (51, 52). Mechanistic investigations reveal that *Ag*TEP1 binding to ookinete surfaces occurs via a multi-phase process: initial rapid association of limited cleaved *Ag*TEP1, followed by SPCLIP1 -facilitated recruitment of uncleaved *Ag*TEP1 for surface deposition, culminating in proteolytic activation by the *Ag*TEP1 enzymatic complex (49).

*Ag*TEP1-triggered immune responses exhibit pathogen size-dependent specialization: phagocytosis for small pathogens (e.g., bacteria) versus melanotic encapsulation for larger invaders (e.g., *Plasmodium*) (36, 53). While *Ag*TEP1 binding is essential for pathogen clearance, its standalone activity proves insufficient, necessitating synergistic interactions with soluble immune co-factors. For instance, studies indicate that even *Ag*TEP1-opsonized ookinetes may evade immune elimination if key co-factors are absent or immunosuppressive molecules like Cap380 are present (49, 54).

Notably, beyond *Ag*TEPs, *Anopheles* mosquitoes possess diverse immune factors including lectins, clip-domain serine proteases (CLIPs), and serine protease inhibitors (serpins) (55, 56). Among these, FREPs—conserved across multiple invertebrates—collaborate closely with TEPs in mollusks like *Biomphalaria* to form pathogen-recognition complexes (e.g., *Bg*FREPs with *Bg*TEPs) (57–59). Whether analogous complexes exist in mosquitoes remains an open question requiring further investigation.

While *Ag*TEP1 has been extensively characterized, the biological functions of other *Ag*TEP members (*Ag*TEP2–19) remain largely unexplored. We have compiled the structural features and immunological functions of *AgTEP* members reported in the current literature into **Table 2**. Future research must systematically elucidate their expression regulation networks, biological roles, and interactions with immune pathways to fully unravel the complexity of *Anopheles* immunity and identify potential intervention targets.

**Table 2:** Structural and functional diversity of the *An. gambiae* TEP gene family: immune roles in pathogen defense, complement activation, and reproductive modulation.

TEP	Key structural notes	Verified/proposed immune roles	Reference
*Ag*TEP1	Gene ID: 36518491; 8 MG domains + CUB + TED (GCGEQ); secreted 165 kDa glycoprotein	Opsonises *Plasmodium* ookinetes, bacteria and fungi; triggers melanisation, phagocytosis, complement-like lysis; stabilised by LRIM1/APL1C; also required for male fertility	(50, 60)
*Ag*TEP2	Gene ID: 3291704; Canonical MG1–8-CUB-TED; GCGEQ preserved	Strongly up-regulated after bacterial challenge or mosGILT knockout; putative broad-spectrum anti-microbial factor (functional proof pending)	(61–63)
*Ag*TEP3	Gene ID: 1275865; ≈1430 aa; TED-MG8 interface and cleavage site conserved	Forms LRIM1/APL1C complex; RNAi oocyst load and blocks periostial haemocyte aggregation; restricts *P. yoelii*/*P. berghei*	(64–67)
*Ag*TEP4	Gene ID: 1278910; “Short-insert” TED; more open TED-MG8 interface	Silencing *P. falciparum*/*P. berghei* infection; cooperates with TEP1/3 in haemocyte clustering and bacterial encapsulation	(65, 68)
*Ag*TEP5; *Ag*TEP6	*Ag*TEP5 Gene ID: 1275867; *Ag*TEP5 Gene ID: 1278851; TE domain present; architecture similar to TEP1	Recognise *Plasmodium* surface proteins; interact with LRIM1/APL1C; act synergistically with TEP1	(63, 69)
*Ag*TEP8	Gene ID: 3291179; expression 3.3-fold 24 h post-mating	Implicated in post-mating immune modulation	(70)
*Ag*TEP9	Gene ID: 1271132; Potential LRIM1/APL1C partner	RNAi alters mosquito susceptibility; may interface with APL1 family members	(66, 71)
*Ag*TEP12	Gene ID: 1275504; Chromosome 3R; divergent clade	No protective effect in assays; transcript in *P. falciparum*-infected heads	(66, 72)
*Ag*TEP13	Gene ID: 1277572	Transcript in infected heads while TEP1, suggesting complementary roles	(72)
*Ag*TEP14 and *An. Stephensi* ^※^	Gene ID: 1277613; TED present	Wolbachia-responsive; silencing had no effect on parasite load (possible redundancy)	(68)
*Ag*TEP15	Gene ID: 1277615; TED (GCGEQ) + CD109/A2M domains	Up-regulated by Wolbachia/*P. yoelii*; RNAi oocysts & induces melanisation via negative regulation of TEP1 and IMD pathway	(36, 68)
*Ag*TEP19	Gene ID: 1271131; 3.3-fold post-mating	Putative role analogous to TEP8	(70)

**※** *A. stephensi* is phylogenetically distinct from the ‘*gambiae* complex’; genetically, the two taxa exhibit a cousin-like relationship—both belonging to the subgenus *Cellia*.

All members of the *Ag*TEP1–19 family are secretory proteins that lack the α2-macroglobulin-type “trap-valve” β-sheets but retain C3-like MG-CUB-TED folds; none exhibit mammalian complement auxiliary domains such as ANA or C345C.

## Schistosomiasis and the snail intermediate host

**Figure 3:**
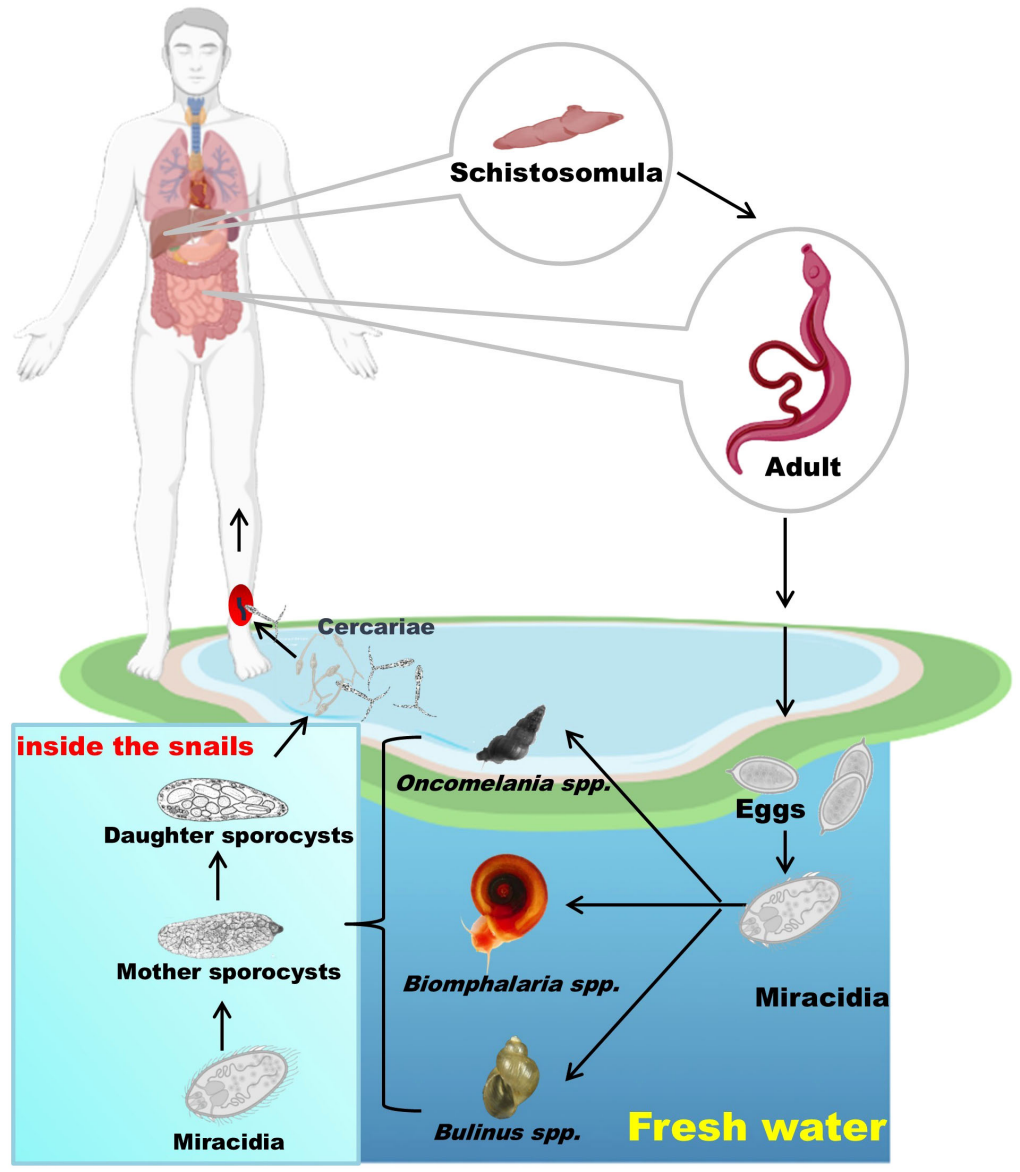
The schistosome life cycle with humans as the definitive host. Eggs released by adult worms hatch into miracidia, which infect snails and develop into cercariae. These are released into water and infect humans, where the worms mature and reproduce. Parts of the materials used in the images within this article originate from BioRender, which we have further modified and enhanced.

Schistosome*s* have a life cycle involving a snail host, and a definitive vertebrate host, which can be a mammal or bird depending on the species (73). They primarily utilize aquatic or amphibious freshwater snails as intermediate hosts to complete the development of larval stages through asexual reproduction, and then undergo sexual reproduction within the definitive host. Here, we provide a brief description of the schistosome life cycle (**Figure 3**). Adult schistosomes, parasitic in many mammals including humans, produce eggs through sexual reproduction (74). Depending on the parasite species, these eggs penetrate the intestinal wall or bladder and are excreted in feces or urine (75). Once outside the host, the eggs hatch under suitable conditions of temperature, light, and osmolarity, giving rise to miracidia (76, 77). The miracidia, equipped with cilia on their surface, can freely swim. When they encounter the appropriate intermediate host snail (such as *Oncomelania* spp. for *S. japonicum*, *Biomphalaria* spp. for *S. mansoni*, and *Bulinus* spp. for *S. haematobium*), they penetrate the snail’s skin and initiate their development within the snail host (73). If the snail is susceptible to the parasite, they undergo development into mother sporocysts, which then produce daughter sporocysts, ultimately leading to the formation of cercariae that are released into the water by penetrating the snail’s tissue (78). When humans or other mammals come into contact with water containing cercariae, they may become infected. The cercariae penetrate the skin and enter subcutaneous veins, where they transform into schistosomula (79). They are then carried by the bloodstream to the right heart chamber, transported to the lungs, and subsequently, through the blood circulation, return to the left heart chamber, entering the arterial circulation (80). Finally, they settle in the mesenteric veins (for *S. japonicum* and *S. mansoni*) or the pelvic venous plexus (for *S. haematobium*), where they mature into adult worms capable of sexual mating and egg production (80).

## TEP-mediated immune response in *B. glabrata* snail

Although there has been extensive research on the biology, pathology, and molecular biology of schistosomes and schistosomiasis, studies on the immunology of the intermediate snail hosts remain relatively limited (81). So far, whole-genome sequencing and annotation have been reported for *B. glabrata* (a critical intermediate host for *S. mansoni*) and *Bulinus truncatus* (an intermediate host for *S. haematobium*), providing important reference resources for investigating the immune interactions between schistosomiasis and intermediate snail hosts (11, 82). Among them, *B. glabrata* has emerged as a significant model organism for studying the interactions between pathogen and hosts, and its immune system has been extensively studied for decades, yielding fruitful research outcomes (83–87). Most invertebrates have a fluid called “hemolymph” in their body cavities, and the diversity of soluble hemolymph proteins is closely associated with the host’s anti-schistosome capabilities (10). This includes various immune molecules involved in anti-schistosome responses, such as *Bg*FREPs (10, 88, 89), lectins (88, 90), *Bg*TEP (17, 91), Biomphalysin (92), Toll-like receptors (*Bg*TLR) (93), granulins (*Bg*GRN) (85), and macrophage migration inhibitory factor (*Bg*MIF) (86). Among them, *Bg*TEP is a key immune component.

*Bg*TEP1 in *B. glabrata* was initially identified by Bender et al. in 1992, revealing its proteinase inhibition activity (94). More recently, *Bg*TEP1 was identified in the study of immunoprecipitates of surface molecules between *Bg*FREP and *S. mansoni* sporocysts (91). Subsequent research has found that *Bg*TEP1 plays an essential role in the recognition and response to epitopes of *S. mansoni*, making it an indispensable immune molecule in the context of anti-parasite infection (17, 40). At present, genomics and proteomics have identified 11 *Bg*TEP proteins (95). Based on the classification similar to the aforementioned TEP superfamily, these 11 *Bg*TEPs can be divided into four branches as follows: (1) complement-like factors (*Bg*C3-1, *Bg*C3-2, and *Bg*C3-3), (2) α-2-macroglobulin (*Bg*A2M), (3) macroglobulin complement-related proteins (*Bg*MCR1 and *Bg*MCR2), and (4) iTEP/CD109 molecules (*Bg*TEP1, *Bg*TEP2, *Bg*TEP3, *Bg*TEP4, and *Bg*CD109) (95). The structural features and immunological functions of *Bg*TEPs in *B. glabrata* are summarized in **Table 3**.

**Table 3:** Functional and evolutionary diversity of the *B. glabrata* TEP family: Complement-like pathways, schistosome defense, and effector complex formation.

TEP/cluster	Key structural notes	Confirmed/proposed immune roles	Reference
*Bg*C3-1; *Bg*C3-2; *Bg*C3-3;	C3-like scaffold; MG domains + thioester	Marks *S. mansoni* sporocysts; initiates complement-like phagocytosis or encapsulation	(40, 95)
*Bg*A2M	α2-macroglobulin homolog with thioester	Traps pathogen proteases; complex cleared via receptor-mediated endocytosis	(17)
*Bg*MCR1; *Bg*MCR2;	Macroglobulin complement-related; thioester absent or partial	Functions under investigation—likely pattern recognition or immune regulation	(95)
*Bg*TEP1	iTEP/CD109-like; secreted	Forms complex with *Bg*FREPs and Biomphalysin; drives ROS-dependent killing of sporocysts; recruits haemocytes	(10, 17)
*Bg*TEP2-4; *Bg*CD109;	iTEP/CD109 family members	Expression patterns suggest immune roles; detailed functions pending	(40, 95)

The cleavage of *Bg*TEP1 is not a prerequisite for pathogen binding. A series of studies have shown that *Bg*TEP1 can bind to the surfaces of different microorganisms and parasites in either full-length or processed forms (17). The binding of *Bg*TEP1 to different developmental stages of *S. mansoni* varies. In the early stage, when miracidia hatch from eggs, *Bg*TEP1 binds in its full-length form, although weakly. The cleaved form also binds to miracidia, but only within the first 3 hours. *Bg*TEP1 also binds to primary sporocysts, predominantly in its full-length form, though cleaved forms are more abundant on sporocysts than on miracidia (17). After binding to invading *S. mansoni* sporocysts, *Bg*TEP1 promotes the recruitment of other subtypes of haemocytes, enabling them to carry out further phagocytosis or encapsulation reactions.

A 2010 study by Mone et al. identified *Bg*TEP1, *Bg*FREP2, and Schistosoma mansoni polymorphic mucins (SmPoMucs) in the precipitate after mixing *B. glabrata* plasma with *S. mansoni* (91). *Bg*FREPs are a class of soluble lectins synthesized and secreted by snail haemocytes. They partially determine the snail’s resistance phenotype against *S. mansoni* (84, 96), primarily by mediating immune recognition of the invading miracidia and sporocyst stages, subsequent clearance responses, and play a crucial role in the immune system of snails (34, 86, 97). Since 1979, it has been known that *B. glabrata* possesses “immune memory” or “acquired resistance” (98), with *Bg*FREPs being linked to this phenomenon (84). The diversity of *Bg*FREPs is thought to result from adaptive evolution. According to the polymorphic compatibility hypothesis, pathogens evolve diverse antigens to evade the immune system, prompting the host to develop a broader set of receptors to identify and eliminate these threats (91, 99). This resembles how vertebrate antibodies recognize a variety of antigens. Each *B. glabrata* snail seems to have a unique *Bg*FREP repertoire, which highlights the importance of *Bg*TEP1 in immune receptor recognition of glycoprotein antigens. Although the role of TEP proteins in mosquitoes and fruit flies has been well studied, it wasn’t until Mone et al.’s research that the function of *Bg*TEP1 in *B. glabrata* became evident. Similar to vertebrate complement C3, *Bg*TEP1 may play a comparable role in the immune system of *B. glabrata*, triggering complement-like pathways.

**Figure 4:**
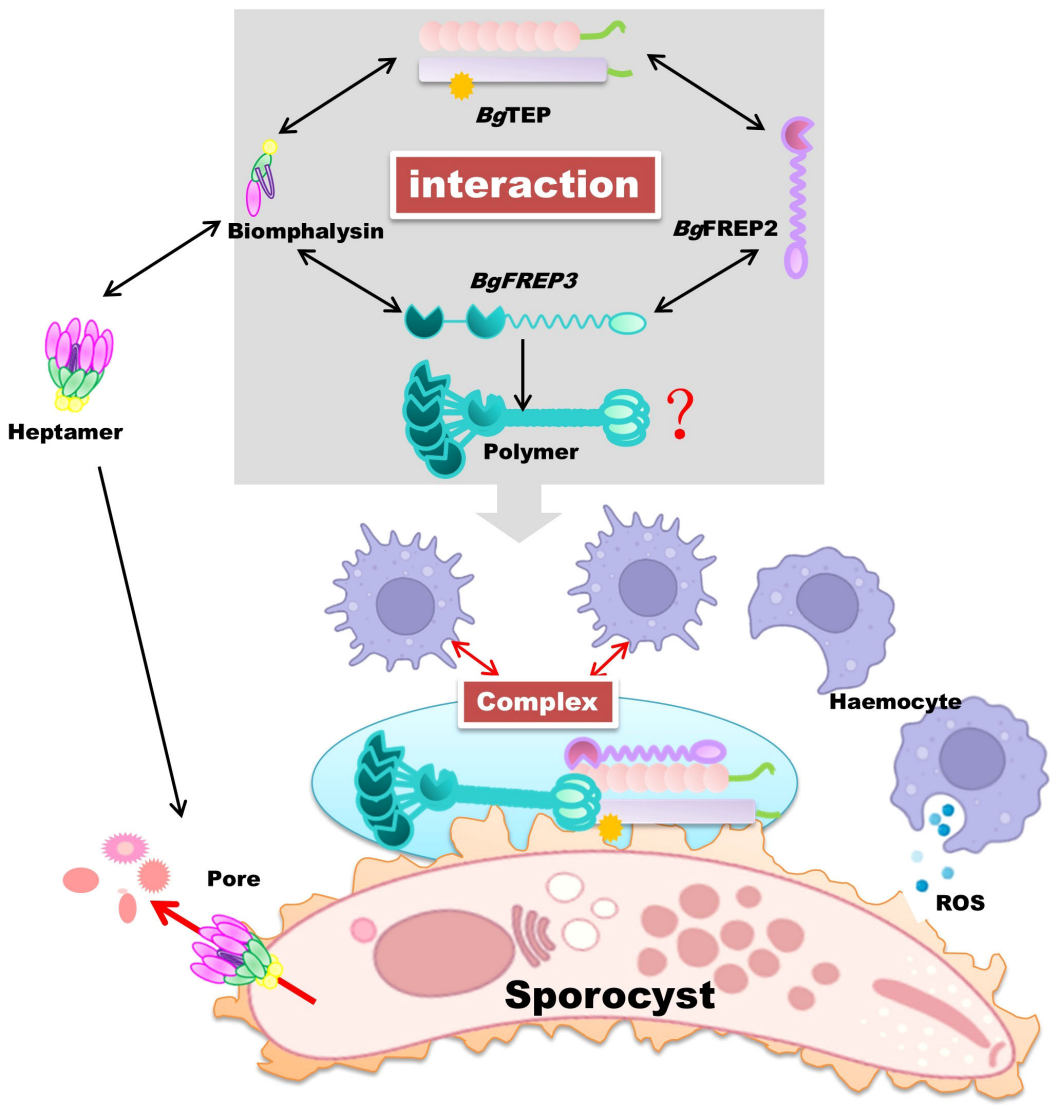
*Bg*TEP as a synergistic immune pathogen eliminator in *B*. *glabrata* snails. In *B*. *glabrata* snails, *Bg*TEP closely interacts with other essential immune proteins, including *Bg*FREP, *Bg*FREP2, and Biomphalysin, to effectively eliminate pathogens in a collaborative manner. Biomphalysin, after forming a heptameric structure, creates pore channels on the surface of invading pathogens, disrupting osmotic balance and ultimately causing their demise. On the other hand, *Bg*TEP, *Bg*FREP, and *Bg*FREP2 form a complex and transmit immune signals to blood cells through unidentified receptors. This transformation converts blood cells into phagocytic subtypes, boosting the secretion of cytotoxic substances, mainly ROS. Together, these synergistic effects effectively eradicate invading parasitic pathogens. The red question marks indicate that although we know *Bg*FREP3 exists as a homomultimer, we are not certain about the mechanism of its multimer formation. We hypothesize that this process may involve protein-protein interactions, post-transcriptional modifications (such as phosphorylation), or the formation of disulfide bonds. Future studies could explore the role of chaperones, conduct mutagenesis analysis, or even perform structural studies to further uncover the specific mechanism of *Bg*FREP3 oligomer formation. Parts of the materials used in the images within this article originate from BioRender, which we have further modified and enhanced.

In 2020, it was further discovered that *Bg*FREP3, *Bg*FREP2, and *Bg*TEP1 interact to form a unique immune complex (illustrated in **Figure 4**). This complex imparts the ability to kill *S. mansoni* sporocysts to haemocytes derived from susceptible snails, nearly equivalent to the haemocytes of resistant snails (10). This sporocyst killing ability can be abolished by ROS scavengers, indicating the crucial role of ROS as effector molecules (10). Based on this study, the *Bg*FREP–*Bg*TEP immune complex is proposed to bind to the pathogen and interacts with a specific receptor on the surface of snail haemocytes, a signal is transmitted to the interior of the cell, initiating an immune response that boosts the synthesis of cytotoxic substances (ROS) to ultimately eliminate the pathogen (10). Despite these findings, the identity of the receptor remains elusive. We speculate it may be a Toll-like receptor, but further research, including co-immunoprecipitation and CRISPR knockouts, is required to confirm this hypothesis (100–102). The interactions between *Bg*FREPs and *Bg*TEP1 in *B. glabrata*’s immune response are critical for mediating effective anti-schistosome defenses, but still not fully understood. Both proteins exhibit pathogen-binding and opsonization capabilities (17, 84, 87, 91). Within their *Bg*FREP–*Bg*TEP immune complex, it is challenging to precisely determine which protein binds directly to the pathogen and which one interacts with the proposed receptor. Immunofluorescence studies have revealed that both *Bg*FREP3 and *Bg*TEP1 can independently bind to the external surface of *S. mansoni* sporocysts, but *Bg*FREP2 requires *Bg*TEP1 to bind effectively to the parasite’s surface (10). This observation prompts questions about the notable disparities in pathogen recognition between *Bg*FREP3 and *Bg*FREP2, both members of the *Bg*FREP family. Notably, *Bg*FREP3 contains two immunoglobulin superfamily (IgSF) domains and forms homomultimers in *B. glabrata* plasma, whereas *Bg*FREP2 lacks multimerization capabilities (10). Although the exact mechanism behind *Bg*FREP3 multimer formation is still unclear, it is speculated that this occurs through the coiled-coil region of the IgSF domain (103). Nevertheless, this hypothesis lacks experimental evidence, and the possibility of *Bg*FREP multimer formation being mediated by the fibrinogen-like (FBG) or IgSF domain cannot be completely disregarded (103). These structural distinctions may elucidate why *Bg*FREP2 requires *Bg*TEP1 to execute its pathogen recognition function. Further research is essential to unravel the intricate mechanisms governing their cooperative roles in the snail’s immune system and explore the potential implications for pathogen defense.

In addition to the aforementioned interaction between *Bg*TEP and *Bg*FREP3 and *Bg*FREP2, a previous study identified an immune interaction between *Bg*TEP1 and Biomphalysin in snail hemolymph in a pull-down experiment using *Bg*TEP1 as bait (10) (**Figure 4**). Biomphalysin, a β-pore-forming toxin (β-PFT), plays a key role in the snail’s immune defense by disrupting the membrane integrity of *S. mansoni*, resulting in the parasite’s lysis (92). While β-PFTs are typically used by bacteria to invade host cells, Biomphalysin in *B. glabrata* is a potent anti-parasitic factor, directly contributing to the destruction of *S. mansoni* (92).

**Figure 5:**
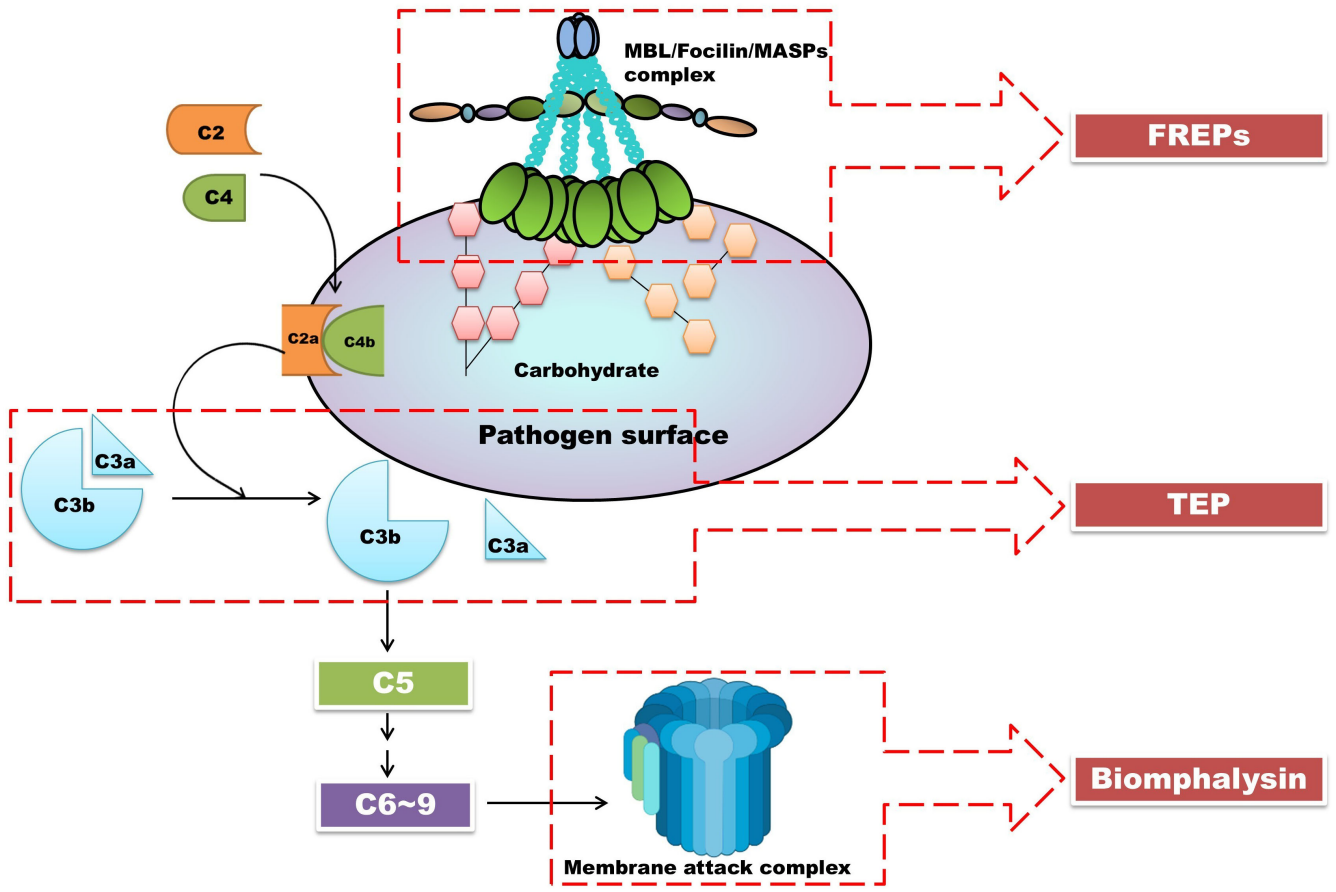
The interactions between *Bg*TEP, lectin-like molecules *Bg*FREPs, and pore-forming toxin Biomphalysin may be the products of the evolutionary process of the lectin pathway. In this figure, we depicted the lectin pathway in vertebrates. Close interactions were found between *Bg*TEP, lectin-like molecules *Bg*FREPs, and pore-forming toxin Biomphalysin in *B*. *glabrata* snails. These correspond to important components of the lectin pathway in vertebrate complement systems. Parts of the materials used in the images within this article originate from BioRender, which we have further modified and enhanced.

*Bg*TEP1 is similar to the human complement C3 protein, which, in the human complement system, leads to the formation of a membrane attack complex (MAC) that disrupts pathogen membranes (1), causing osmotic imbalance and cell death. Both Biomphalysin and MAC form pore-like structures on cell membranes, resulting in cell lysis. This suggests that the interaction between *Bg*TEP1 and Biomphalysin may serve a similar function in *B. glabrata*, resembling the role of the complement system in humans. Furthermore, ongoing research has suggested potential parallels between the immune factors identified in *B. glabrata* snails and the important members of the lectin pathway (**Figure 5**). Although the parallels are not perfect, it outlines a rough pathway: *Bg*FREPs correspond to pathogen recognition parts, such as ficolin and Mannose-Binding Lectin, *Bg*TEP1 corresponds to complement C3 protein, and Biomphalysin confers to MAC’s action. This suggested model provides valuable clues for a deeper understanding of the evolution and function of the immune system.

## Comparative analysis of TEPs in *Anopheles* and *Biomphalaria*

Despite belonging to evolutionarily distant phyla, *An. gambiae* (arthropod) and *B. glabrata* (mollusk), their TEPs exhibit functional convergence in innate immunity. These TEPs universally play central roles in host defense by recognizing, opsonizing, and eliminating invading pathogens, albeit through distinct operational contexts and associated mechanistic frameworks summarized in **Table 4**.

**Table 4:** Comparison of *Ag*TEP1 and *Bg*TEP1: Structural Features, and Immune Functions in *An. gambiae* and *B. glabrata*.

Feature	AgTEP1 (An. gambiae)	BgTEP1 (B. glabrata)
Immune Targets	*Plasmodium* parasites (e.g., *P. falciparum*) (104) Bacteria and fungi (45)	*S. mansoni* sporocysts and miracidia (10, 91) Bacteria and fungi (17)
Activation Mechanism	Proteolytic cleavage by unknown proteases (43) Thioester bond hydrolysis for pathogen binding (42)	Cleavage not required for pathogen binding Full-length and cleaved forms can bind (10, 91)
Stabilization Factors	LRIM1/APL1C heterodimer stabilizes cleaved *Ag*TEP1 (104) Prevents premature activation (42)	Involve interactions with *Bg*FREPs (10, 91)
Pathogen Recognition	Binds to *Plasmodium* ookinetes via thioester-dependent and independent mechanisms (43, 91)	Binds to *S. mansoni* sporocysts and miracidia via *Bg*FREP interactions (10)
Immune Response	Phagocytosis of bacteria (50) Melanization and encapsulation of *Plasmodium* parasites (104)	Recruitment of haemocytes for phagocytosis or encapsulation (91) Direct killing by ROS (10)
Evolutionary Context	Homologous to vertebrate complement factors C3/C4/C5 (42) Primitive complement system	Similar to vertebrate complement C3 May represent an intermediate evolutionary stage (10, 91)

Both *Ag*TEP1 and *Bg*TEP1 harbor a highly conserved GCGEQ thioester motif, enabling covalent binding to pathogen surfaces post-activation to function as opsonins, thereby inducing phagocytosis, encapsulation, or other immune clearance mechanisms (17, 105). Additionally, both require binding to co-factors for stability and functional enhancement: *Ag*TEP1 relies on the LRIM1/APL1C complex, while *Bg*TEP1 cooperates with *Bg*FREPs and Biomphalysin to exert immune effects (10, 22).

Functionally, both TEP systems recognize diverse pathogens, including protozoans, helminths, and bacteria, suggesting that TEPs—as ancient and conserved immune factors—likely represent an evolutionarily conserved core of broad-spectrum immune mechanisms in invertebrates.

Despite structural similarities, *Ag*TEP1 and *Bg*TEP1 exhibit distinct activation pathways. *Ag*TEP1 activation depends on proteolytic cleavage in the hemolymph and stabilization by the LRIM1/APL1C complex, reflecting stringent protein-level regulation (14, 106). In contrast, *Bg*TEP1 is primarily synthesized in haemocytes, with its expression modulated by host genetic background, developmental stage, and environmental stimuli, highlighting transcriptional-level regulation (10, 107).

Their effector pathways also diverge significantly: *Anopheles* predominantly employs melanization responses and complement-like lysis for pathogen clearance, whereas *Biomphalaria* utilizes hemocyte-mediated encapsulation and ROS-dependent extracellular cytotoxicity (108, 109). These mechanistic differences reflect host adaptations to their respective parasites (*Plasmodium* vs. *Schistosoma*), including structural features, survival strategies, and immune evasion tactics. However, it is also possible that other TEP family members in mosquitoes and snails contribute to these immune responses, which may account for some of the observed functional differences.

In *An. gambiae*, ROS also play a crucial role in *Ag*TEP1-mediated immune responses, particularly in melanization. Melanization is a key defense mechanism in the insect immune system, involving the encapsulation of pathogens with melanin to prevent their further spread (110, 111). Research has shown that ROS are essential in melanization, especially during *Ag*TEP1-mediated clearance of *Plasmodium* parasites (54, 60). When *Ag*TEP1 binds to *Plasmodium* ookinetes, ROS production is significantly enhanced, leading to the melanization and death of the parasites (112). This process shares similarities with the ROS generation mechanism mediated by *Bg*TEP1 in *B. glabrata*. In *An. gambiae*, ROS not only directly participate in pathogen killing but also promote melanin synthesis and deposition by activating enzymatic reactions in the melanization pathway (113).

In *B. glabrata*, after *Bg*TEP1 forms an immune complex with *Bg*FREP3 and *Bg*FREP2, it activates the ROS generation pathway in haemocytes. These ROS act as effector molecules, directly attacking *S. mansoni* sporocysts, leading to membrane rupture and death (10). Studies have shown that ROS scavengers significantly reduce the killing ability of *B. glabrata* against *S. mansoni*, further confirming the importance of ROS in this process (10). Additionally, ROS production is closely related to the binding and signaling of *Bg*TEP1. Through interactions with *Bg*FREP3 and *Bg*FREP2, *Bg*TEP1 forms an immune complex that activates the ROS generation pathway in haemocytes. This process resembles the vertebrate complement system, where ROS also serve as critical effector molecules in pathogen clearance (95, 114).

Both *Ag*TEP1 and *Bg*TEP1 exhibit co-adaptive dynamics shaped by host-parasite interactions. For instance, *Ag*TEP1 displays strain-specific responses to different *Plasmodium* isolates across *Anopheles* populations, with activity influenced by host and pathogen genetic polymorphisms (21, 115). Similarly, *Bg*TEP1 expression differs markedly between resistant and susceptible snail strains, with resistant individuals mounting stronger *Bg*TEP1-mediated immune responses during early infection (10, 40).

These findings underscore the pivotal role of TEPs in the long-term evolutionary “arms race” between hosts and parasites, retaining their core structural architecture while evolving highly plastic adaptive functions. For instance, enhancing *Ag*TEP1 expression or stability via genetic engineering could significantly reduce *Plasmodium* loads in mosquitoes, thereby interrupting malaria transmission (27, 116). Similarly, targeting the interaction between *Bg*TEP1 and *Bg*FREPs may bolster snail resistance to *Schistosoma*, effectively disrupting the schistosomiasis transmission cycle (10).

Notably, in *B. glabrata*, the *Bg*TEP1-Biomphalysin interaction generates lytic pore-forming complexes that directly induce *S. mansoni* sporocyst lysis (10, 117). While analogous MAC-like structures remain unconfirmed in *Anopheles*, this discovery provides critical insights into TEP-mediated immune mechanisms across hosts. Compared to mosquito strategies countering *Plasmodium* motility, the specific responses mediated by *Ag*TEP1 and *Bg*TEP1 suggest that *Biomphalaria*’s defense against sessile *Schistosoma* larvae emphasizes reactive oxygen species (ROS) and perforin-like complexes, reflecting possible divergent adaptations within individual TEP molecules across species (49, 118).

Furthermore, in *B. glabrata*, *Bg*FREP2 and *Bg*FREP3 synergize with *Bg*TEP1 to form immune complexes that convert susceptible snails into partially resistant phenotypes (91). In contrast, although *Anopheles* FREPs participate in *Plasmodium* clearance as recognition receptors (114), no direct FREP-TEP interaction has been documented; *Ag*TEP1 functionality remains dependent on LRIM1/APL1C stabilization (14, 119). These findings suggest that boosting co-factor expression or developing small-molecule mimics to stabilize TEP complexes could enhance immune efficacy, enabling genetic or ecological interventions to block malaria and schistosomiasis transmission (120).

In summary, the TEP system not only occupies a pivotal position in invertebrate immune evolution but also provides a theoretical and practical foundation for innovative disease control. By unraveling the functional and mechanistic intricacies of TEPs in *Anopheles* and *Biomphalaria*, we may develop precision interventions targeting these two major parasitic diseases.

## Research gaps and future perspectives

Despite significant progress in elucidating the roles of TEPs in vector immunity, several key gaps remain that hinder a comprehensive understanding of their function and application potential.

Current research is heavily concentrated on *Ag*TEP1 in *An. gambiae* and *Bg*TEP1 in *B. glabrata*, leaving the majority of other TEP family members understudied. In *An. gambiae*, over a dozen *Ag*TEPs have been identified, yet their individual or synergistic roles in immune defense remain poorly defined (27, 36). Similarly, *B. glabrata* likely possesses a broader repertoire of TEP-like genes, but functional validation is lacking. Expanding the functional annotation of these paralogs through CRISPR/Cas9, RNAi, and proteomics will be essential for uncovering hidden immune networks (121).

The molecular triggers and regulatory pathways governing TEP activation remain only partially understood in both species. For instance, while proteolytic cleavage is a known activation mechanism in *Anopheles*, the upstream signals initiating this process, and their modulation by infection or environmental stressors, remain to be clarified. In *B. glabrata*, the transcriptional regulation of *Bg*TEP1 in response to parasite infection, pollutants, and other stimuli is only beginning to be explored (54, 103). Future research should focus on delineating the signaling cascades and epigenetic factors that control TEP expression and activity.

The interaction between TEPs and other immune components such as PRRs, ROS, and AMPs is not well defined (36, 122). Given the dynamic nature of innate immunity, TEPs likely operate as part of a broader immune network rather than as isolated effectors (40). Understanding this crosstalk, both in basal conditions and during infection, will provide a more integrated view of host defense strategies (45).

Most TEP studies are conducted under laboratory conditions that may not fully represent natural infection dynamics. Ecological factors such as temperature, microbiota composition, and co-infections can all influence TEP expression and function. Field-based transcriptomic and functional studies are needed to validate laboratory findings and assess the real-world relevance of TEP-mediated responses, especially in disease-endemic areas.

The potential of TEPs as targets for malaria vector-based interventions, such as genetic manipulation or immunostimulation, remains largely theoretical. Future efforts should evaluate whether enhancing TEP function in mosquito or snail populations can reduce parasite development and transmission *in vivo*. Additionally, identifying small molecules or microbial adjuvants that upregulate TEP expression may offer novel avenues for biological control strategies.

## Conclusion

TEPs are central effectors of innate immunity in invertebrate disease vectors, mediating recognition and elimination of a wide range of pathogens. In both *An. gambiae* and *B. glabrata*, TEPs serve as functional analogs to vertebrate complement proteins, operating through conserved thioester motifs to tag pathogens for immune clearance.

While *Ag*TEP1 and *Bg*TEP1 share structural and functional similarities, their activation mechanisms, interacting partners, and effector pathways reflect the distinct evolutionary and ecological contexts of their hosts. These differences highlight the adaptive plasticity of TEP systems and emphasize their role in host–parasite coevolution.

Comparative analysis of TEP-mediated immunity in mosquitoes and snails offers valuable insights into the evolution of invertebrate defense systems and provides a foundation for novel vector-based disease control strategies. By bridging findings across phylogenetically distant taxa, we can better understand how innate immunity has diversified to meet the challenges of parasitic infection.

Future research should continue to explore the complexity, regulation, and translational potential of TEPs, with the goal of leveraging this ancient yet dynamic immune mechanism in the global fight against malaria and schistosomiasis.

## Glossary

ACTs: Artemisinin-based Combination Therapies
AgTEP: Anopheles gambiae Thioester-containing Protein
AMP: Antimicrobial Peptide
An. gambiae: Anopheles gambiae
An. stephensi: Anopheles stephensi
APL1C: Anopheles Plasmodium-responsive Leucine-rich Repeat 1C
BgA2M: Biomphalaria glabrata α-2-macroglobulin
B. glabrata: Biomphalaria glabrata
β-PFT: Beta Pore-forming Toxin
BgFREP: Biomphalaria glabrata Fibrinogen-related Protein
BgGRN: Biomphalaria glabrata Granulin
BgMCR: Biomphalaria glabrata Macroglobulin Complement-related Protein
BgMIF: Biomphalaria glabrata Macrophage Migration Inhibitory Factor
BgTEP: Biomphalaria glabrata Thioester-containing Protein
BgTLR: Biomphalaria glabrata Toll-like Receptor
Cap380: Plasmodium oocyst capsule protein 380
CLIP: Clip-domain Serine Protease
CUB: Complement C1r/C1s, Uegf, Bmp1 Domain
FBG: Fibrinogen-like Domain
FREP: Fibrinogen-related Protein
GCGEQ: Conserved Thioester Motif (Gly-Cys-Gly-Glu-Gln)
HPX2: Heme Peroxidase 2
IgSF: Immunoglobulin Superfamily
iTEP: Insect Thioester-containing Protein
LRIM1: Leucine-rich Repeat Immune Molecule 1
MAC: Membrane Attack Complex
MG: Macroglobulin
NOX5: NADPH Oxidase 5
P. berghei: Plasmodium berghei
P. falciparum: Plasmodium falciparum
P. vivax: Plasmodium vivax
P. yoelii: Plasmodium yoelii
PRR: Pattern Recognition Receptor
PZQ: Praziquantel
ROS: Reactive Oxygen Species
S. mansoni: Schistosoma mansoni
s.l./s.s.: sensu lato/sensu stricto
SPCLIP1: Serine Protease-like CLIP-domain Protein 1
TED: Thioester Domain
TEP: Thioester-containing Protein
TLR: Toll-like Receptor.
